# Brain Activity Correlates With Cognitive Performance Deterioration During Sleep Deprivation

**DOI:** 10.3389/fnins.2019.01001

**Published:** 2019-09-19

**Authors:** Hugo F. Posada-Quintero, Natasa Reljin, Jeffrey B. Bolkhovsky, Alvaro D. Orjuela-Cañón, Ki H. Chon

**Affiliations:** ^1^Department of Biomedical Engineering, University of Connecticut, Storrs, CT, United States; ^2^Facultad de Ingeniería Mecánica, Electrónica y Biomédica, Universidad Antonio Nariño, Bogota, Colombia

**Keywords:** electroencephalography, error awareness test, sleep deprivation, performance, reactivity, response inhibition

## Abstract

We studied the correlation between oscillatory brain activity and performance in healthy subjects performing the error awareness task (EAT) every 2 h, for 24 h. In the EAT, subjects were shown on a screen the names of colors and were asked to press a key if the name of the color and the color it was shown in matched, and the screen was not a duplicate of the one before (“Go” trials). In the event of a duplicate screen (“Repeat No-Go” trial) or a color mismatch (“Stroop No-Go” trial), the subjects were asked to withhold from pressing the key. We assessed subjects’ (*N* = 10) response inhibition by measuring accuracy of the “Stroop No-Go” (SNGacc) and “Repeat No-Go” trials (RNGacc). We assessed their reactivity by measuring reaction time in the “Go” trials (GRT). Simultaneously, nine electroencephalographic (EEG) channels were recorded (Fp_2_, F_7_, F_8_, O_1_, Oz, Pz, O_2_, T_7_, and T_8_). The correlation between reactivity and response inhibition measures to brain activity was tested using quantitative measures of brain activity based on the relative power of gamma, beta, alpha, theta, and delta waves. In general, response inhibition and reactivity reached a steady level between 6 and 16 h of sleep deprivation, which was followed by sustained impairment after 18 h. Channels F_7_ and Fp_2_ had the highest correlation to the indices of performance. Measures of response inhibition (RNGacc and SNGacc) were correlated to the alpha and theta waves’ power for most of the channels, especially in the F_7_ channel (*r* = 0.82 and 0.84, respectively). The reactivity (GRT) exhibited the highest correlation to the power of gamma waves in channel Fp_2_ (0.76). We conclude that quantitative measures of EEG provide information that can help us to better understand changes in subjects’ performance and could be used as an indicator to prevent the adverse consequences of sleep deprivation.

## Introduction

This paper explores the effect of sleep deprivation on both cognitive performance and brain activity in healthy subjects. Given that alertness and brain activity are known to diminish in parallel during prolonged periods of wakefulness (Borbély et al., 2016), objective techniques for the assessment of brain activity may enable prevention of some of the common fatal events caused due to human error, and reduce the economic cost of human performance deterioration. For example, performance deterioration due to fatigue can lead to accidents in jobs that frequently require working late at night or for long periods (Leger, 1994; Costa, 1996; Lyznicki et al., 1998). Electroencephalography (EEG) is a widely used method for providing quantitative and better dynamic assessment of the electric activity in the brain (Tatum, 2014). We focus on the relative power of EEG as it has been used to avoid the high variability of the absolute power (Monastra et al., 2001; Kropotov, 2010; Pavlov and Kotchoubey, 2017).

An effective means to monitor human job performance is a long-sought goal, especially ways to detect errors and warn an individual if their ability has been affected by something like sleep deprivation. If detection were possible, the individual could be warned to enact appropriate mitigating countermeasures (Gehring et al., 1993; Ullsperger and von Cramon, 2001). EEG is a powerful candidate for such monitoring, as studies of neural responses to performance errors suggest that the prefrontal and anterior cingulate cortices are critical to error processing, although the specific roles of those regions are a matter of debate (Bush et al., 2000).

It has been shown that both the intensity of EEG waves upon falling asleep and the desire to sleep are linked to the period of time spent awake (Borbély et al., 2016). The process of becoming increasingly tired throughout the day is attributed to the effects on the brain of the neurotransmitter adenosine. Endogenous adenosine inhibits basal forebrain and mesopontine cholinergic neurons, which play a large part in abrupt shifts in EEG frequency, termed EEG arousal (Steriade et al., 1990). As extracellular adenosine concentration increases, so does inhibition of the cholinergic neurons. There are many reasons why researchers believe that adenosine produces the effects of tiredness. During wakefulness, neural metabolism is much higher than during deep sleep, while adenosine concentration in the brain and neural metabolic activity have been linked. Also, caffeine is known to reduce the effects of adenosine and promote arousal in subjects and their EEG waveforms, by blocking adenosine receptors, Porkka-Heiskanen et al. (1997).

The typical EEG waveform consists of alpha, beta, theta, gamma, and delta waves, which differ in frequency as well as amplitude (Dement, 1974; Tatum, 2014). Alpha, beta, and gamma waves are in a higher frequency range (>8 Hz). In most cases, high-frequency low-amplitude waves are concurrent with higher activity of the brain. When awake, most people exhibit beta and alpha wave patterns. Beta waves have the highest frequency and the lowest amplitude than other waves in an awake, alert individual. They are dominant when a person is not sleeping. During periods of relaxation our brain waves become slower, which is accompanied by an increase in the amplitude of alpha waves. They tend to diminish when the eyes are open or when the brain exercises mental effort. In other words, lower levels of activity are represented by higher levels of alpha waves. Gamma waves are linked to voluntary motor movement, learning, and memory. Delta and theta waves are in a lower frequency range. Delta waves are related to slow-wave sleep and have been found during some continuous-attention tasks (Kirmizi-Alsan et al., 2006). As for the theta waves, high amplitude theta waves correlate with memory function while lower-amplitude ones are linked to decreased alertness and increased drowsiness. Low-frequency (delta and theta) brain activity has been observed to increase as a result of sleep deprivation (Cajochen et al., 1999; Hung et al., 2013; Gorgoni et al., 2014; Fattinger et al., 2017).

Several studies have explored the correlation between sleep deprivation, brain activity, and behavioral performance (Lorenzo et al., 1995; Corsi-Cabrera et al., 1996; Forest and Godbout, 2000; Yokoi et al., 2003; Gorgoni et al., 2014; Bernardi et al., 2015). Significant correlations have been found between the EEG increase in theta activity observed during sleep deprivation and a slowing down of reaction times (Gorgoni et al., 2014), or an increase in behavioral errors (Bernardi et al., 2015; Quercia et al., 2018).

Traditionally, EEG signals are divided in short-time epochs and spectral analysis is applied to the epochs to obtain continuous waves. In this study, we have explored a different approach, in which the different waves were isolated in the time domain using finite impulse response (FIR) filters. The spectral power of each wave was computed for each channel in the 4-min segment while the subjects performed a cognitive task, and normalized to the total spectral power of each channel. This analysis quantifies the brain activity during the entire period of time the subject is undergoing a task, instead of linking it directly to specific instantaneous events (e.g., errors or hits), as it is traditionally done. This method of quantifying brain activity can account for the possible relationship between brain activity at a given moment and the committing of future errors. In other words, this method allows us to correlate the overall brain activity with the overall performance, during a given period of time. This approach is simpler and has led us to find stronger correlations between brain activity and performance.

The EEG provides a practical and quantitative way to assess the electrical activity of the brain. This study seeks to examine the correlation between relative power of EEG waves and performance over a period 4 min, during a cognitive assessment. To evaluate this, we have employed a simple yet robust test that requires the subject to maintain response inhibition and reactivity. This information from the neurophysiological measures of the oscillatory activity of the brain can complement physiological measures used in studies focused on autonomic reactions (Posada-Quintero et al., 2017, 2018), to predict the effect of sleep deprivation on human autonomic response and performance.

The resulting data could lead to a tool for prediction of the drowsiness and fatigue states of a person by measuring the electrical activity of the brain. To the best of our knowledge, no other study has analyzed the correlation between task-related relative EEG activity in the different frequency bands and performance during periods of prolonged wakefulness. This information can be used to predict and reduce risk in environments where it is expected that people suffer from sleep deprivation.

## Materials and Methods

### Subjects

Ten healthy volunteers (three females, seven males; age range 25–35) were enrolled in this study. Participants were required to remain awake for at least 24 h. An experiment manager was always observing the participants, to ensure safety and procedural validity of the experimental protocol. During the 7 days prior to the experiment, participants recorded their sleep patterns in a data sheet, to indicate compliance to the experimental constraints.

### Protocol

For participating in the study, the participants were required to halt all consumption of stimulants and depressants beginning 48 h prior to the start of the experiment. Participants were instructed to arrive at the experimental facility, located at the University of Connecticut, within 2 h of waking up on the morning of the study. Participants completed a learning run for the experimental task within the 1st h of arrival. Afterward, they were asked to perform a run of the task every 2 h for the duration of the 24-h period, for a total of 12 runs besides the training run. Participants remained in the building for approximately 25 h to allow for the completion of all 12 runs and training.

**FIGURE 1:**
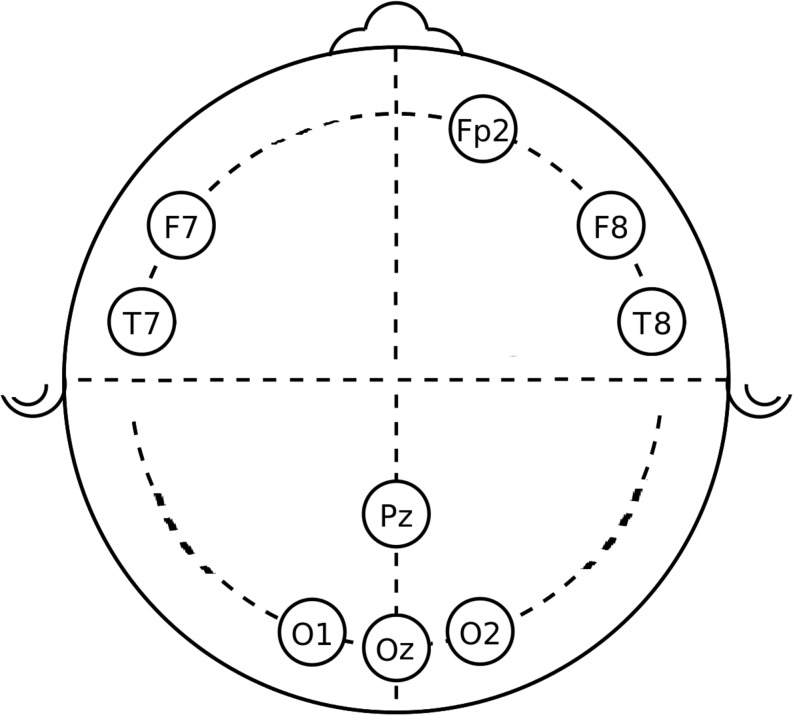
Location of EEG channels recorded. The circles represent the approximate location of the electrode for each channel.

Twenty minutes before starting the task, an EEG cap with ten electrodes was placed on the participant’s head. We used an actiCHamp amplifier (Brain Products GmbH, Gilching, Germany) with an EasyCap electrode system (EasyCap GmbH, Herrsching-Breitbrunn, Germany). The electrodes were placed in frontal (F), frontal-polar (Fp), temporal (T), parietal (P), and occipital (O) positions, as shown in Figure 1. Electrode gel was used to provide appropriate conductance between the electrode and the skin. Prior to EEG data measurement, the electrodes’ impedance was checked to make sure it was lower than 5 kΩ, to ensure good electrode connection to the scalp. Nine EEG channels were recorded: Fp_2_, F_7_, F_8_, O_1_, Oz, Pz, O_2_, T_7_, and T_8_, while the subject was performing the error awareness task (EAT) with their eyes open. The input signals were referenced to the ears, band pass filtered with cutoff frequencies of 0.5 and 50 Hz, and digitized at a sampling rate of 200 Hz.

Between runs, leads were disconnected and the subjects were allowed to eat or do any activity other than exercising or sleeping (for example, using a laptop, reading, going to the restroom, talking on the phone, and so forth). To avoid any undesirable influence on sympathetic arousal, subjects had to remain in the building. To ensure adherence to the protocol, food was provided. This study was carried out in accordance with the recommendations of the Institutional Review Board of the University of Connecticut, and with their approval of the study protocol. All subjects gave written informed consent in accordance with the Declaration of Helsinki.

### Performance Assessment

**FIGURE 2:**
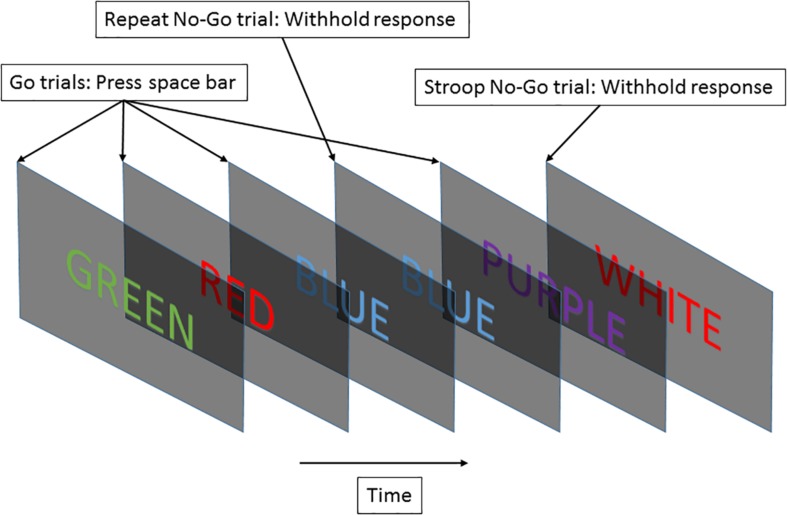
The EAT required subjects to respond to a stream of color names by pressing a key, and to withhold their response based on “No-Go” trial rules.

For this study, we used the EAT. We presented a serial stream of names of colors in colored fonts. Each name was presented for 900 ms, with 600 ms between names. The test was 5 min long in this study. Participants were trained to respond to each new screen with the space bar. Subjects were requested to press the space bar for “Go” trials and withhold this response for “No-Go” trials. A “Go” trial occurred when the font color matched the color’s name and did not fall into the category of a “No-Go” trial. A “No-Go” trial could be generated by two different circumstances: (1) if the same word was presented on two consecutive trials (repeat “No-go” task), or (2) if the color named did not match the font color (Stroop “No-Go” task) (Hester et al., 2005). Figure 2 illustrates the procedure. The second instance of “No-Go” trial represents a Stroop effect, which induces cognitive stress in the subject (Stroop, 1935). The two withhold conditions were meant to maintain subjects’ vigilant attention so they did not fall back on repetitive behavior. Constantly having to monitor the color match kept subjects alert.

To assess subject performance, the accuracy of Stroop and Repeat “No-Go” tasks (SNGacc and RNGacc, respectively) and average “Go trial” reaction times (GRT) were computed for every trial. SNGacc and RNGacc constitute measures directly proportional to how vigilantly a subject paid attention. For its part, GRT is a simple measure to assess subject reactivity. Notice that GRT exhibits an inverse relationship to subject performance, as a higher reaction time represents lower performance.

### EEG Data Processing

Four minutes of clean EEG data were extracted for each subject during each EAT trial. During EAT, subjects sat in a chair and were instructed not to talk or move. For this reason, the EEG signals were stable and no significant artifact corruption was usually observed. When a few instances of motion artifacts were identified, we removed the corrupted data segments (a fraction of a second) from all the channels. The data segment removed was compensated for by selecting a larger window, so that all the analyzed segments were 4 min long. To assess the brain activity, we computed quantitative measures based on spectral analysis of the nine channels of EEG data. We used five bands, with ranges set at <4 Hz (delta), 4–8 Hz (theta), 8–13 Hz (alpha), 13–30 Hz (beta), and >30 Hz (gamma) in accordance with the literature. The five waves were obtained using FIR filters designed using the Parks-McClellan optimal equiripple approach. The power was computed using the following equation:

$${\text{P}}_{x}=\frac{1}{L}\,\sum_{t=1}^{L}x^{2}\,\left(t\right)$$

Where *x* is the signal and *L* is the length of the signal (4 min). This is equivalent to the mean square of the signal. We used the relative power (to the total power) of these waves as a quantitative measure of brain activity.

### Statistics

A total of 45 measures of brain activity were computed—the power of the five bands in each of the nine channels of EEG. As a measure of subject performance, average GRT, and Stroop No-Go and Repeat No-Go accuracies (SNGacc and RNGacc, respectively) were computed for every run of every subject.

To evaluate the significance of the differences in the measures of EEG and performance during the experiment, we performed multiple comparisons of the indices between the 12 runs. The normality of each index was tested using the one-sample Kolmogorov-Smirnov test (Massey, 1951; Miller, 1956; Marsaglia et al., 2003). Given that all measures were normally distributed, the one-way analysis of variance (ANOVA) was performed to test for significant differences between runs. The Bonferroni method was used for correction of multiple comparisons.

### Correlation Analysis

We computed the Pearson’s correlation coefficient (r) between mean values of measures of performance (GRT, SNGacc, and RNGacc) and the 45 measures of brain activity (from EEG) of the participants (over the 12 runs of the 24-h test). The *t*-test was used to assess the statistical significance of the correlation coefficient (the null hypothesis was that the product moment correlation coefficient was zero) (Spiegel, 1961).

## Results

**FIGURE 3:**
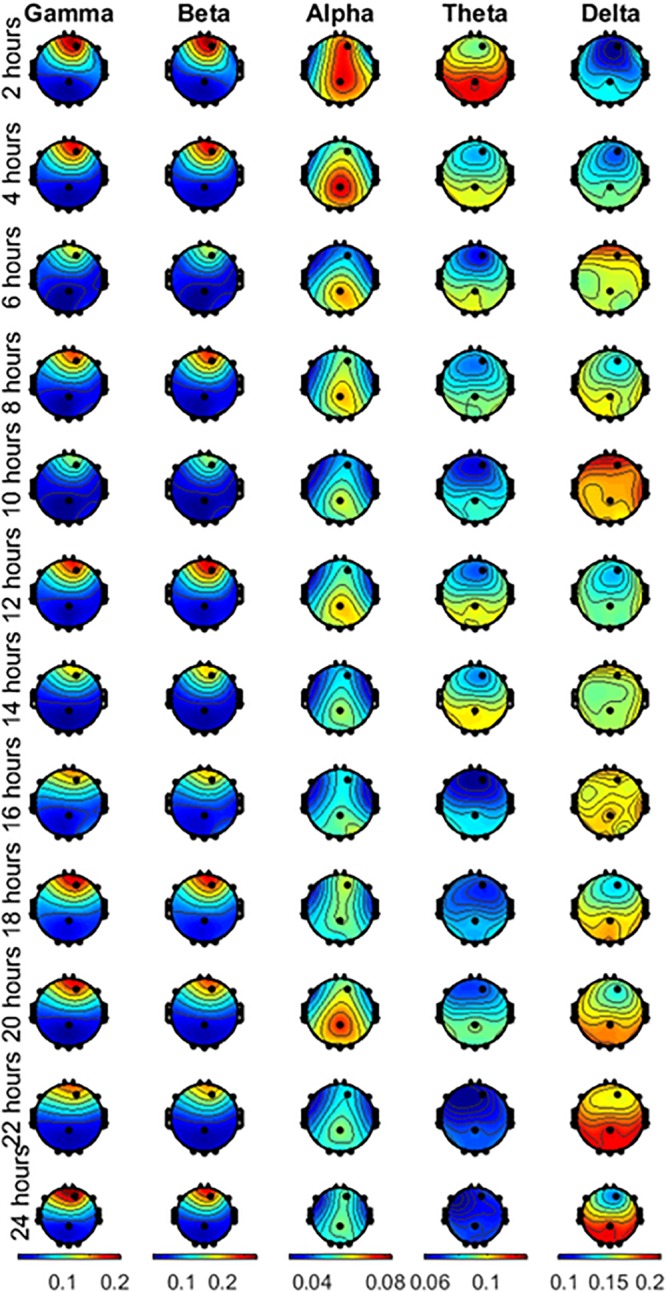
Averaged scalp topographies of the gamma, beta, alpha, theta and delta waves of the subjects performing the EAT for the 12 runs during the 24 h of testing.

The time duration between the waking time and the first session was less than 40 min. This short time duration allows us to expect that possible circadian effects are negligible because of the small difference in the start time of the experiment across subjects. The mean starting time of the first session was 8:25 AM. Figure 3 shows the plots of the topographic maps of the scalp data field in a 2-D circular view (looking down at the top of the head) using interpolation on a fine Cartesian grid. Note that each wave is plotted in a different scale, shown at the bottom of the figure. The rows represent the hours of sleep deprivation at the time of the measurement, and the columns represent the EEG waves considered in this study. The topographic maps are the result of averaging all the subjects together. In this study, gamma and beta waves were more prominent in the front throughout the 24 h. Alpha waves were more prominent in the parietal region, with noticeable changes during the 24 h of the experiment. Theta waves exhibited higher power in the parietal, occipital, and temporal regions, with an apparent reduction noticeable after 16 h. Delta waves presented higher power in the parietal, occipital, and temporal regions than it did in the other lobes and their power increased toward the end of the 24-h period.

**FIGURE 4:**
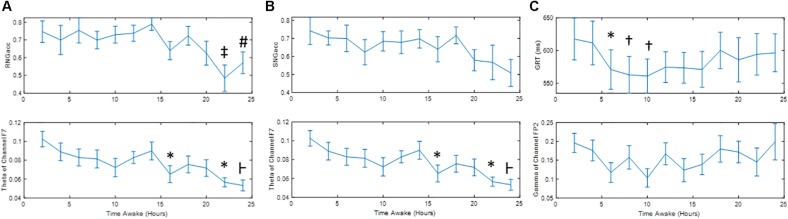
Mean ± Standard Error of **(A)** RNGacc, **(B)** SNGacc, and **(C)** GRT measures and their most-correlated quantitative measure of EEG. Symbols denote significant difference: ^∗^ to run 1; ⊢ to runs 1, 2 and 7; ‡ to runs 1, 3, 4, 5, 6, 7, 9; # to run 7; and † to runs 1 and 2.

**FIGURE 5:**
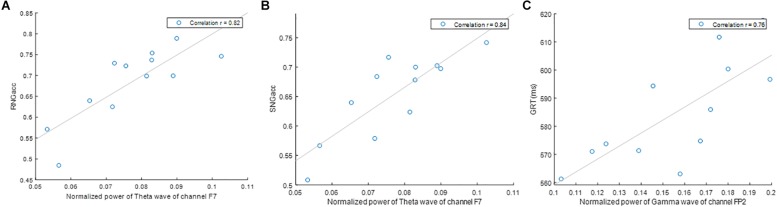
Correlation of **(A)** RNGacc and power of theta wave of channel F7, **(B)** SNGacc and power of theta wave of channel F7, and **(C)** GRT and power of gamma wave of channel T8.

Figure 4 shows the change in performance measures during the 24 h of sleep deprivation, along with the quantitative measure of EEG with which they exhibited the highest correlation. Significant differences between the runs are marked. Figure 5 shows the correlation plot for those pairs of measures. Figures 4A,B show that SNGacc and RNGacc indices exhibited stable values for a majority of the 1st 14 h of the experiment, followed by a sustained drop in value after 18 h. RNGacc showed a significant decrease at 22 h compared to most of the runs before 18 h, and a decrease at 24 h compared to the first 14 h runs. RNGacc exhibited a peak value at 14 h. GRT at 6, 8, and 10 h was significantly lower than the measurement at 2 h. GRT increased after 14 h. GRT can be summarized as 4 h of learning period after which the subjects reached a plateau where the average reaction time stabilized between 6 and 16 h, ending in a final increase in the reaction time, possibly related to subjects’ tiredness.

Using quantitative analysis of EEG data, we performed correlation analysis between brain activity and performance. Table 1 includes the results for the correlation analysis of all quantitative measures of EEG available in this study (five waves times nine channels), to the three measures of performance obtained from the subjects performing the EAT. Higher overall correlation to the brain activity was found for the SNGacc and RNGacc, compared to GRT. The highest correlation was found between the SNGacc measure and the theta waves of the F_7_ channel (*r* = 0.84). Such wave exhibited the highest correlation to the RNGacc (*r* = 0.82). RNGacc and SNGacc were found to be significantly correlated with theta waves of all the channels except the frontal polar (Fp_2_). These measures were also found to be inversely correlated to delta waves in all lobes except for frontal (F) channels and the frontal polar channel (Fp). As for the gamma and beta waves, RNGacc and SNGacc were only found to be significantly correlated to those waves in frontal channels (F). These two measures of performance were not found to be correlated to alpha waves of any channel.

**TABLE 1:** Correlation (*r*) between EEG measures and performance measures.

		**Performance measure**		
		**RNGacc**	**SNGacc**	**GRT**
**EEG channels and waves**				
**FP_2_**	Gamma	–0.24	–0.20	0.76^∗^
	Beta	0.02	0.08	0.7^∗^
	Alpha	0.09	0.22	0.71^∗^
	Theta	0.50	0.47	0.51
	Delta	–0.05	–0.09	−0.76^∗^
**F_7_**	Gamma	−0.75^∗^	−0.72^∗^	0.39
	Beta	−0.63^∗^	−0.58^∗^	0.46
	Alpha	0.10	0.22	0.64^∗^
	Theta	0.82^∗^	0.84^∗^	0.18
	Delta	–0.30	–0.40	−0.62^∗^
**F_8_**	Gamma	−0.63^∗^	−0.59^∗^	0.19
	Beta	−0.6^∗^	–0.55	0.20
	Alpha	–0.18	–0.12	0.49
	Theta	0.65^∗^	0.64^∗^	0.17
	Delta	–0.18	–0.27	–0.50
**O_1_**	Gamma	–0.32	–0.20	0.30
	Beta	–0.15	0.02	0.36
	Alpha	0.16	0.23	0.41
	Theta	0.73^∗^	0.73^∗^	0.17
	Delta	−0.8^∗^	−0.83^∗^	–0.10
**O_z_**	Gamma	–0.46	–0.31	0.55
	Beta	–0.23	–0.03	0.56
	Alpha	0.30	0.34	0.33
	Theta	0.73^∗^	0.73^∗^	0.14
	Delta	−0.76^∗^	−0.8^∗^	–0.14
**Pz**	Gamma	–0.38	–0.25	0.53
	Beta	–0.22	–0.07	0.56
	Alpha	0.30	0.34	0.39
	Theta	0.72^∗^	0.7^∗^	0.15
	Delta	−0.8^∗^	−0.82^∗^	–0.17
**O_2_**	Gamma	–0.06	–0.12	–0.49
	Beta	0.15	0.12	–0.50
	Alpha	0.41	0.39	0.00
	Theta	0.74^∗^	0.74^∗^	0.15
	Delta	−0.81^∗^	−0.82^∗^	0.10
**T_7_**	Gamma	–0.33	–0.26	0.33
	Beta	–0.16	–0.05	0.36
	Alpha	–0.15	–0.08	0.46
	Theta	0.71^∗^	0.71^∗^	0.18
	Delta	−0.74^∗^	−0.82^∗^	–0.26
**T_8_**	Gamma	–0.34	–0.17	0.68^∗^
	Beta	–0.21	–0.02	0.74^∗^
	Alpha	0.03	0.16	0.73^∗^
	Theta	0.69^∗^	0.69^∗^	0.20
	Delta	–0.56	−0.66^∗^	–0.50

*^∗^*p* < 0.05.*

The highest absolute correlation of GRT was with the gamma and delta waves of channel Fp_2_ (0.76). GRT was significantly correlated to gamma, beta, and alpha waves of channels T_8_ and Fp_2_. This measure of performance was also found to be moderately correlated to alpha and delta waves of channel F_7_.

## Discussion

We have implemented a task that allows simultaneous evaluation of response inhibition and reactivity, as components of overall cognitive performance of subjects undergoing sleep deprivation. This test has been used previously to study the relationship between autonomic reactions and performance on young and healthy subjects (Posada-Quintero et al., 2017). In this study, we have used quantitative measures of EEG for the assessment of oscillatory brain activity. Results allowed us to observe the changes on the different waves (covering different frequency ranges) during the 24 h of sleep deprivation, and how the power of those waves correlates with changes in the measures of response inhibition and reactivity. This led to the conclusion that these quantitative measures of EEG provide valuable information that could potentially be used to prevent an individual from inadvertently performing undesirable actions when sleep deprived.

Remarkably, two channels contained oscillatory power that was the most correlated to the indices of performance: channels F_7_ and Fp_2_. Specifically, the power of the theta waves of channel F_7_ exhibited the highest correlation to RNGacc (*r* = 0.82) and SNGacc (*r* = 0.84), related to subjects’ response inhibition. The power of the gamma and delta waves of channel Fp_2_ exhibited the highest correlation to the measure of reactivity, GRT (*r* = 0.76). This is an interesting observation because if a reduced set of channels of EEG is sufficient for providing a neurophysiological objective measure that is able to track the effects of sleep deprivation, it increases the feasibility of deploying the EEG for practical applications.

In this study, theta waves exhibited high correlation with the “No-Go” trials, assessed with the RNGacc and SNGacc measures. Theta waves have been linked to a subject’s voluntary repression of provoked responses, as they have been found to increase in circumstances where individuals are intentionally trying to inhibit a reaction (Kirmizi-Alsan et al., 2006). Our findings corroborate this relationship between subjects’ inhibitory responses and theta waves, particularly high in the frontal channels.

Beta waves are linked to intense brain activity, stress, active thinking, and focus, among other circumstances (Lalo et al., 2007; Baumeister et al., 2008). The power of the beta waves in the frontal channels was moderately correlated to subjects’ sustained response inhibition (RNGacc and SNGacc measures). This behavior of power of the beta waves suggests that subjects’ active thinking and focus were diminished after 14 h of sleep deprivation, and it might have contributed to the reduction in performance related to vigilant attention.

We found that gamma waves in the frontal polar (Fp_2_) and right temporal (T_8_) channels were significantly correlated to subjects’ reaction time (GRT) throughout the 24-h experiment. Gamma waves in frontal channels (F_1_ and F_2_) were related to subjects’ vigilant attention (RNGacc and SNGacc). Gamma waves are present during short-term memory matching of recognizable elements presented to the sight, and other sensory stimuli (Kisley and Cornwell, 2006; Kanayama et al., 2007). With some controversy, these waves are thought to be implicated in the conscious perception (Gray, 1999; Vanderwolf, 2000). We observed that the power of the gamma waves mostly correlated to the rapidness the subjects exhibited in making reaction decisions, which involves memory, matching, and motor functions.

Alteration of the power in the delta waves observed in this study (Figure 3). was inversely correlated with the RNGacc and SNGacc measures (beyond −0.81), mainly in the occipital, parietal, and temporal channels (especially O_1_, O_2__,_ and P_Z_). They were also inversely correlated to GRT in the frontal polar channel (−0.76). The measure of reactivity, GRT, seemed to be more linked to the delta oscillatory activity of the right side of the brain, as higher correlation was found in channel T_8_.

As for the alpha waves, it seems like they moved from the frontal to the parietal lobe during the experiment. Alpha waves are reportedly found predominantly in posterior sides of the head, and are reduced with drowsiness and sleep (Palva and Palva, 2007). Alpha waves are also linked to inhibitory control (Kamarajan et al., 2004; Klimesch, 2012). Alpha waves only exhibited significant correlation to GRT in channel Fp_2_ (−0.71).

Performance and physiology are expected to be as sensitive to circadian rhythms as to sleep deprivation in a 24-h period of wakefulness. However, sleep deprivation is known to cause an overall increase in reaction time and increased errors of omission and commission (Lim and Dinges, 2008). By the end of this study, at about 8 AM, the effect of the circadian rhythm should, in theory, cause a recovery of the subject’s responsiveness and response inhibition. Instead, Figure 4 shows a continued trend of increase in the GRT and decrease in response inhibition measures (RNGacc and SNGacc). This indicates that participants are more affected by sleep deprivation than by the circadian rhythm. The same interpretation can be made with the measures of brain activity.

**FIGURE 6:**
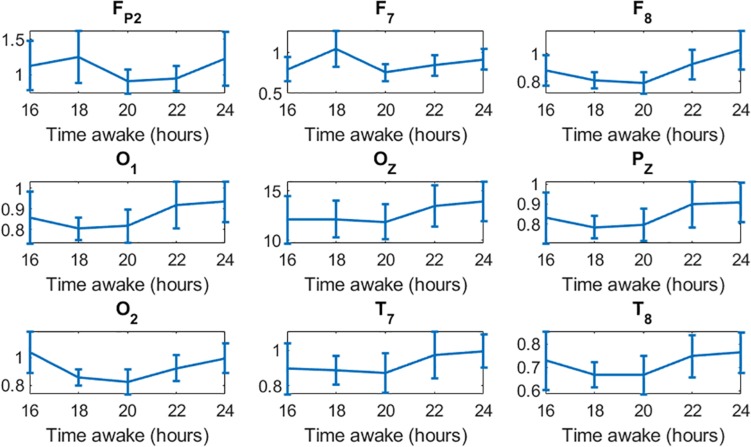
Increase in the absolute power of theta waves in all channels toward the end of the 24-h experiment.

Multiple studies have reported that low-frequency waves, mainly theta waves, increase as a result of sleep deprivation (Cajochen et al., 1999; Hung et al., 2013; Gorgoni et al., 2014; Fattinger et al., 2017). There is also evidence of a moderate positive correlation between theta waves and the committing of errors (Lorenzo et al., 1995; Corsi-Cabrera et al., 1996; Forest and Godbout, 2000; Yokoi et al., 2003; Gorgoni et al., 2014; Bernardi et al., 2015). In those studies, the power of the waves was normalized to the power of the first run. As we were interested in studying the changes in the waves over increasing time of sleep deprivation, and the correlation of the overall brain activity to performance, we did not normalize to the power in the first run to avoid changing the “system gain” for each subject. Instead, for quantifying the power of the bands we used the relative power of the bands with respect to the total power. We hypothesize that this difference in approach may have led to different trends in theta waves. Although we found a strong positive correlation between the power of delta waves and the impairment of performance (maximum *r* = 0.81), we found a strong negative correlation between the power of theta waves and performance (maximum *r* = −0.84). Note that delta waves exhibited much higher power (ranging 0.1–0.22) compared to theta waves (ranging 0.06–0.15) (Figure 3). As delta waves increase by the end of the experiment, it produces an overall decrease in the relative power of theta waves. Figure 6 shows the absolute power of theta waves normalized to the power of the first run, from 16 to 24 h of sleep deprivation. The expected increase in theta waves as a result of sleep deprivation concomitant with the impairment of performance is also observed in our data. In spite of the different analytical approach (absolute vs. relative power), our results are substantially in line with previous findings indicating that sleep deprivation is associated with a relative increase of low-frequency (vs. high-frequency) activity that is in turn correlated with performance impairment.

The limited sample size and characteristics of the set of participants (ten healthy subjects) limit the conclusiveness of this study. This was a quite demanding study protocol requiring 24 h of sleep deprivation. To draw general conclusions, more data are needed. We acknowledge that given the many correlations relevant for the study, there is a good chance that random data would yield significant correlation (*p* < 0.05). The analysis of gender differences is not possible with the current data set and needs to be explored in future studies. Also, EAT measures of performance are certainly linked to reactivity and attention, but do not fully replicate the kinds of tasks a subject encounters in real life. It should be noted that despite the unavoidable variability between subjects’ skills and training conditions, and a limited sample size, we found considerable consistency in measures of performance throughout the ten subjects.

## Conclusion

Alteration of the power of theta and beta waves in frontal channels followed a trend similar to measures of response inhibition, measured by accuracy in performing the EAT, whereas the changes of power in gamma and delta waves in the frontal polar channel resembled more the changes in reactivity, measured by reaction time. We conclude that quantitative measures of EEG provide information to better understand changes in subject performance and could be used to prevent the adverse consequences of sleep deprivation. Furthermore, objective neurophysiological measures can provide valuable information to systems intended to assess a human’s readiness to perform.

## Data Availability Statement

The datasets generated for this study are available on request to the corresponding author.

## Ethics Statement

This study was carried out in accordance with the recommendations of the Institutional Review Board of The University of Connecticut, with written informed consent from all subjects. All subjects gave written informed consent in accordance with the Declaration of Helsinki. The study protocol was approved by the Institutional Review Board of The University of Connecticut.

## Author Contributions

HP-Q performed the experiments, data analysis, and wrote a draft of the manuscript. NR and JB performed the experiments and edited the manuscript. AO-C performed the data analysis and edited the manuscript. KC conceptualized the study and edited the final version of the manuscript.

## Conflict of Interest

The authors declare that the research was conducted in the absence of any commercial or financial relationships that could be construed as a potential conflict of interest.
